# LiDAR Sensor Parameter Augmentation and Data-Driven Influence Analysis on Deep-Learning-Based People Detection

**DOI:** 10.3390/s25103114

**Published:** 2025-05-14

**Authors:** Lukas Haas, Florian Sanne, Johann Zedelmeier, Subir Das, Thomas Zeh, Matthias Kuba, Florian Bindges, Martin Jakobi, Alexander W. Koch

**Affiliations:** 1 IFM—Institute for Driver Assistance Systems and Connected Mobility, Kempten University of Applied Sciences, Junkerstraße 1A, 87734 Benningen, Germany; 2 Institute for Measurement Systems and Sensor Technology, Technical University of Munich, Theresienstr. 90, 80333 Munich, Germany; 3 Faculty of Electrical Engineering, Kempten University of Applied Sciences, Bahnhofstraße 61, 87435 Kempten, Germany; 4 Blickfeld GmbH, Barthstr. 14, 80339 Munich, Germany

**Keywords:** deep learning, LiDAR sensor, neural networks, people detection, point cloud, point cloud augmentation

## Abstract

Light detection and ranging (LiDAR) sensor technology for people detection offers a significant advantage in data protection. However, to design these systems cost- and energy-efficiently, the relationship between the measurement data and final object detection output with deep neural networks (DNNs) has to be elaborated. Therefore, this paper presents augmentation methods to analyze the influence of the distance, resolution, noise, and shading parameters of a LiDAR sensor in real point clouds for people detection. Furthermore, their influence on object detection using DNNs was investigated. A significant reduction in the quality requirements for the point clouds was possible for the measurement setup with only minor degradation on the object list level. The DNNs PointVoxel-Region-based Convolutional Neural Network (PV-RCNN) and Sparsely Embedded Convolutional Detection (SECOND) both only show a reduction in object detection of less than 5% with a reduced resolution of up to 32 factors, for an increase in distance of 4 factors, and with a Gaussian noise up to μ=0 and σ=0.07. In addition, both networks require an unshaded height of approx. 0.5 m from a detected person’s head downwards to ensure good people detection performance without special training for these cases. The results obtained, such as shadowing information, are transferred to a software program to determine the minimum number of sensors and their orientation based on the mounting height of the sensor, the sensor parameters, and the ground area under consideration, both for detection at the point cloud level and object detection level.

## 1. Introduction

In addition to the application field of highly automated driving, light detection and ranging (LiDAR) sensors are increasingly being used to count people in publicly accessible places or the field of security applications [1,2]. In these applications, it is important to reliably detect people in the sensor’s working area and still be as cost-effective as possible. Edge devices are often used for this purpose, where data processing is carried out locally on the device itself [3]. For easy installation, they must be especially energy-efficient. Neural networks are usually used to detect people. In order to design the entire sensor system as efficiently as possible, both the requirements for the optical sensor of the LiDAR and the required computing power of the entire edge device should be optimized. For this purpose, the influences of different LiDAR sensor parameters on the resulting object detection with deep neural networks (DNNs) must be investigated, as in [4] for cameras. These relationships are usually not analytically realizable for DNNs and their black box characteristics [5] and thus have to be treated in a data-driven approach. Therefore, this paper analyzes the correlation between sensor and sensor setup parameters and object detection using various DNNs to define future optimization potential. For this purpose, the dependence of the distance between the scene and the sensor, the resolution of the sensor, and the effect of noise and shading in the point cloud on DNN-based people detection are investigated.

The remainder of this paper is organized as follows. Section 2 discusses the related work on point cloud augmentation and LiDAR-based people detection using deep learning. The augmentation of the point clouds with the resolution, distance, noise, and shading parameters are explained in Section 3. Section 4 shows the data set structure used, and in Section 5, the deep learning methods for the training and testing are described. The effects of the augmented parameters on LiDAR-based object detection are discussed in Section 6. The work of this paper is summarized in Section 8, and an outlook is given in Section 9.

## 2. State of the Art

In the field of artificial neural networks (ANNs) based on image data from cameras, the influence of different camera effects, such as on the accuracy of the camera system itself and, as in [6], on the prediction performance of object recognition algorithms and DNNs, was investigated. In [7], the authors investigate the influence of noise and brightness on the performance of camera-based DNNs for object detection. For LiDAR data, the authors of [8] show the differences between image and point cloud object detection and the influence of distant objects on object detection in point clouds. In order to overcome the influence of different point densities at different distances, the authors in [9] present a method in which the point density of objects to be detected is evenly distributed over the entire point cloud. However, the point density for object detection is influenced by different sensors’ distance and resolution. This is why the authors in [10,11] are working on making DNNs robust to interchanging data from different LiDAR sensors with different resolutions. Sensor-specific effects also influence the quality of object detection with DNNs, as shown in [12], based on the example of the motion distortion effect. Another important challenge in developing measurement systems used with deep learning methods and developing deep learning methods themselves is the lack of data. For this reason, the authors of [13] present a measurement setup for automatically generating a labeled data set for people detection. In [14,15], the authors present a method for the variation in the pose of the point cloud for developing robust deep learning methods. The authors in [16] present a method for generating new synthetic point clouds by interpolating existing point clouds. In [17], new synthetic point clouds are generated by combining existing point clouds of rotating LiDAR sensors along the azimuth axis and tilting the inserted sections. This allows the data set to be artificially expanded, and the developed DNNs are more transferable to other measurement setups. Augmenting existing point clouds is another way of artificially enlarging data sets and recreating certain effects [18]. In [19,20,21,22], methods for augmenting point clouds with a reduced resolution are presented. Therefore, in [19], the point cloud is divided into different cubes, and random points in these cubes are removed. In [20], a position in the 2D view of the point cloud is randomly selected, and all points in a random radius around this position are removed. The authors of [21] present a method in which the key points of the point cloud are identified and removed using adversarial learning. In [22], edges are defined as key points from which only a few points are removed, and surfaces as zones from which more points can be removed. In [23], the shape of objects is distorted based on a vector field to better generalize object detection with DNNs. This is also done by testing the sensitivity to noise. To do this, the authors add or delete critical points from the point cloud to generate noise. In [24,25], the point clouds are augmented by noise. In [24], the Cartesian coordinates of the points are randomly noisy. The authors of [25] augment the homogeneous coordinates of each point of the point cloud with noise based on a uniform distribution.

However, none of these papers follow a hardware-oriented approach in which the resolution and noise of the point cloud are augmented according to the operation principle of the hardware of the real LiDAR sensor.

This paper presents a functional principle-based augmentation of sensor and measurement setup parameters based on LiDAR point clouds for people detection. For this purpose, specific parameters of real measured point clouds for people detection are presented after considering the fundamentals—the sensor and measurement setup. Sensor-internal parameters, such as noise in the angle measurement of the mirrors and depth measurements and a lower resolution of the scan pattern in both azimuth and elevation direction, are augmented close to the functional principle of the hardware. The mounting height and the shadowing of people by other objects are also augmented and discussed. The influences of individual parameters on the performance of DNNs for object detection are then presented. Based on the acquired results, a software tool for optimizing LiDAR-based measurement setups for people detection is introduced. To the best of the authors’ knowledge, this is the first work that applies such functional principle-based sensor parameter augmentations to real measured point clouds for people detection and quantifies their influence on the object detection performance of DNNs. In the following sections, the functionality of the sensors used is described in more detail. Therefore, this paper analyzes the correlation between sensor and sensor setup parameters and object detection using various DNNs to define future optimization potential. For this purpose, the dependence of the distance between the scene and the sensor, the resolution of the sensor, and the effect of noise and shading in the point cloud on DNN-based people detection are investigated.

### 2.1. ToF LiDAR Sensors

LiDAR sensors measure the distances of reflected light beams in space to detect objects. The LiDAR sensors emit laser pulses. The emitted laser pulses hit obstacles in the sensor’s scanning range, the so-called field of view (FoV); they are partially reflected and detected in the sensor. For so-called time of flight (ToF) LiDAR sensors, the ToF between the emission of the light pulse from the sensor and the re-arrival of the reflected pulse at the sensor’s detector is measured. To determine the radial distance *r* between the location of the reflection and the sensor, this is calculated from the speed of light *c* and the ToF [26], as shown in Equation (1).

(1) $$r=\frac{c\;\cdot \;ToF}{2}$$

LiDAR sensors vary in size and performance depending on their area of application. However, strict safety standards must be observed when used in the vicinity of people, as the sensor’s laser operates in a wavelength range that is invisible to humans. Therefore, the eye’s natural protective mechanisms are limited. To ensure safety and prevent eye injuries, the average transmitting power of the laser is therefore limited [27]. This energy limitation makes it difficult to detect the received light power at the sensor detector, which is reduced by diffuse reflections through the atmosphere, partial absorption on the object, and non-directional backscattering. Without any effect on the average transmission power, the laser can be focused more, increasing the energy density. However, there is a risk that small spatial angles and homogeneous surfaces will result in total reflection of the light beam. For this reason, high-performance detectors must be used [28] to obtain detections on the point cloud level in challenging environments and large distances. In addition to the ToF and the output angles of the laser beam, the relative velocity of an object to the sensor can also be determined and the intensity of the returning light measured [28,29]. Different ToF LiDAR sensor types control the laser pulse’s direction in various ways. These different scanning mechanisms are presented below.

### 2.2. Qb2 Operating Principle

**Figure 1:**
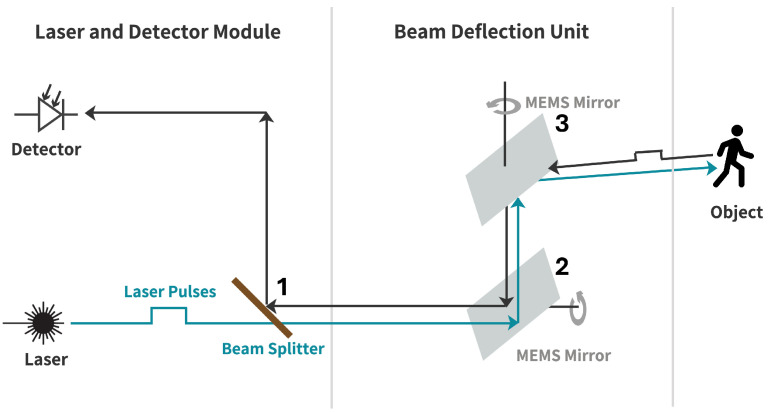
Working principle of the Qb2 LiDAR sensor [26].

The Qb2 from Blickfeld GmbH (Munich, Germany) is used in this paper because it provides the data structure described in [13] for the automated label toolchain used and has a verifiable scanning pattern. The Qb2 is a s microelectromechanical system (MEMS)-based ToF LiDAR sensor, whose structure and operating principle are shown schematically in Figure 1. The laser passes through a beam splitter (1) and is emitted in the desired direction via two MEMS-based mirrors (2, 3). The two mirrors perform oscillating rotational movements to generate a defined scan pattern [26]. The distance and angle measurements performed are saved in the form of point clouds.

**Figure 2:**
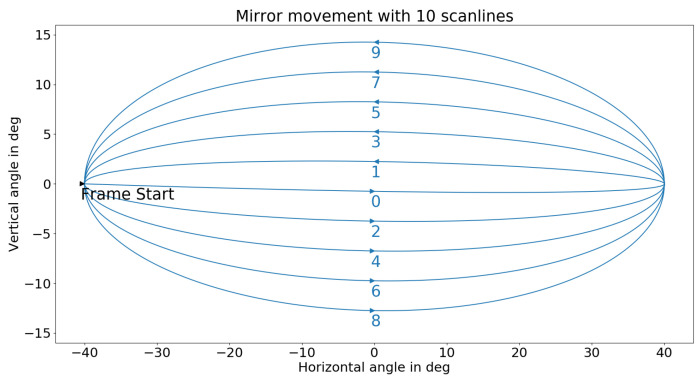
Visualization of the scan pattern of the Qb2 LiDAR sensor from Blickfeld GmbH [30].

By oscillating the mirrors (2, 3) in Figure 1, the sensor generates an elliptical scan pattern, as shown in Figure 2.

### 2.3. Artificial Neural Networks for 3D Object Detection

Like the brain as its natural counterpart, ANNs consist of individual neurons, whereby the complexity of nature with about $10^{11}$ nerve cells of a human brain and an estimated $10^{14}$ connections of artificial applications remains unmatched to date [31]. The artificial neurons have inputs and outputs and are usually organized in different layers. An output value is generated from an input vector using an activation function and passed on via weighted connections. This creates an information flow from the neurons in the input layer via one or multiple inner layers to the output nodes. The learning method forms the basis of the ANNs, as the weighting of the connections between the nodes is adjusted according to its scheme during training. These adjustments make it possible to reproduce output values for identical and similar inputs and thus achieve a learning capability [32]. There are various training methods for ANNs. The supervised training used for this work requires labeled data. This means that a correct result is already available as a label, and the ANN’s output is compared with this during training. The resulting error is then minimized. Other learning methods include unsupervised training—for example, pattern recognition—and reinforcement learning, in which no error is calculated between the actual and desired output, but only feedback on success or failure is transmitted [33].

#### 2.3.1. Feedforward Neural Networks

Feedforward neural networks (FNNs) form one category of ANNs. These networks only have connections from one layer to the following layer. FNNs are fully connected if each neuron has a connection to each neuron in the next layer. There are also derivatives in which connections skip one or more layers [34].

#### 2.3.2. Convolutional Neural Networks

Convolutional neural networks (CNNs) are FNNs with limited connections between the layers, primarily designed for image processing. The pixel values of an image distributed across the input layer are grouped into small subsets to identify structures that emerge later in the network. Therefore, input values are folded into convolution layers with filter kernels. The results of these operations are then reduced in pooling layers before the information is further processed in hidden layers of an FNN [35]. Classical 2D-based CNNs perform worse with 3D data than 2D image processing, so these models must be specially adapted for 3D applications. One method is to project the point clouds onto a plane to obtain different 2D views [36]. Another common practice is dividing the data into so-called voxels, smaller three-dimensional cubes representing the points in the volume. Although this variant is computationally efficient, it also entails a loss of information within the voxels [37]. The two CNNs used in the course of this work are presented below. These are specially optimized for 3D object detection with LiDAR point clouds and are based on the basic concept of CNN.

#### 2.3.3. SECOND

**Figure 3:**
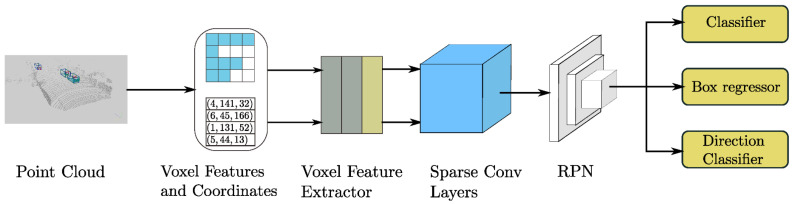
Structure of the SECOND model ([36], S. 4).

Sparsely Embedded Convolutional Detection (SECOND) uses voxels, as described previously, for object detection. The coordinates and the number of points per cube are recorded during creation. After extracting features of the voxels from the individual points using an FNN, as shown in Figure 3, spatially sparse convolution is used. This means that only the voxels containing data points are considered in a CNN. With many free volumes in a 3D data set, SECOND remains computationally efficient. Finally, the points are analyzed with a regional proposal network (RPN), which creates so-called regions of interest (ROIs). In these, the exact positions of objects are determined, and corresponding bounding boxes are created [36].

#### 2.3.4. PV-RCNN

**Figure 4:**
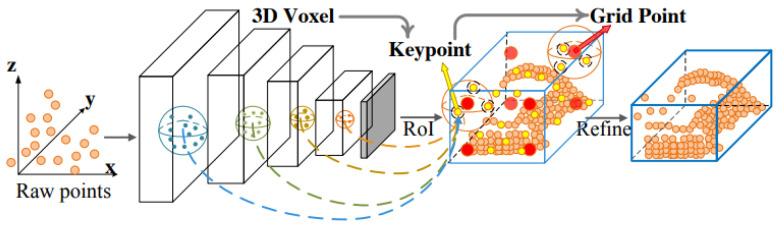
Structure of the PV-RCNN model ([37], S. 1).

The PointVoxel-Region-based Convolutional Neural Network (PV-RCNN) also creates a defined number of voxels from the point clouds. Apart from processing these voxels using sparse convolution and the creation of ROIs, as in the SECOND model, point-based methods are additionally used. Key points are extracted from the voxels using furthest point sampling. This means that points as far apart as possible within a cube are selected to represent its features. Further information about the ROI is then extracted from these key points. This use of the two different approaches, illustrated in Figure 4, ensures high accuracies of PV-RCNN in object detection [37].

### 2.4. Validation Metrics

To evaluate the results of the DNNs with the different variations in the augmented data sets, result parameters are required that illustrate the performance of the people detection models. As in [38], a threshold value for the Intersection over Union (IoU) is used from which an object is considered a true positive. As per Equation (2), this is calculated as the quotient of the IoU set of the predicted bounding boxes (predBB) and the labels (gtBB) [38].

(2) $$IoU=\frac{predBB\cap gtBB}{predBB\cup gtBB}$$

Precision is the rate of the correct predictions (TruePositives) of the neural network compared to the total number of predictions (TruePositives+FalsePositives). This can be calculated formally using Equation (3) [38].

Recall is the rate of the correct predictions (TruePositives) compared to the total number of labeled objects (TruePositives+FalseNegatives). The recall can be calculated using Equation (4) [38].

(3) $$Precision=\frac{True\;Positives}{True\;Positives+False\;Positives}$$

(4) $$Recall=\frac{True\;Positives}{True\;Positives+False\;Negatives}$$

The average precision (AP) is calculated, as described in [39], from the area under the precision and recall curve. The AP can be calculated from the precision *P* and recall *R* according to the following Equation (5):

(5) $$AP=\int_{0}^{1}P\left(R\right)\;dR$$

For the calculation of the mean intersection over union (MIoU) of the bounding boxes, the mean value of the IoU for the number of bounding boxes *n* predicted by the neural network is calculated according to the following Equation (6).

(6) $$MIoU=\frac{1}{n}\sum_{i=1}^{n}IoU$$

The values for recall, AP, and an average of the predicted number of objects were output as implemented in [40] according to [38,39]; the further metrics were implemented according to the requirements of this work.

## 3. Augmentation of the LiDAR Dataset

**Figure 5:**
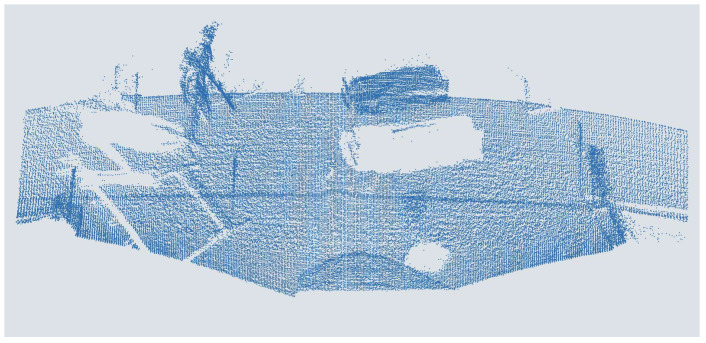
Person in a point cloud of a MEMS-based scanning ToF LiDAR sensor.

DNNs for LiDAR object detection are usually trained using supervised learning. During training, the deviation between the prediction of the network and the ground truth is calculated. After each training, a batch is used to adapt the weights of the neurons in the ANN via backpropagation. A labeled data set is required for this. Therefore, the data set presented in [13] is used for the people detection considered in this work. This data set was generated with the LiDAR sensor Qb2 from the Blickfeld GmbH [41] and contains 14,819 labeled point clouds. An exemplary point cloud is shown in Figure 5. The sensor was mounted stationary on the ceiling of the entrance area of the canteen at Kempten University of Applied Sciences at a height of approximately 3.8 m, which is almost 3.5 m wide and around 7.0 m long. The objects appearing in the LiDAR point clouds are mostly people walking or standing and various objects of different sizes that have been placed or moved through. At the edges of the Qb2’s FoV, parts of the door frames and a wall can be seen. Labels were automatically generated using the object lists from reference cameras mounted in the room. The labels are stored as .txt files in the unified 3D box label format [30].

In order to investigate the influences of the LiDAR sensor parameters resolution, distance, noise, and shading on people detection with DNNs, the influences of the sensor parameters on the point cloud must be represented in the respective data set. In order to implement this as time and cost-efficiently as possible, these effects are augmented onto the existing real data set. This way, the data set can be built on existing data containing real scenes and people. Augmentation allows large amounts of data to be generated very quickly. A corresponding generation with real hardware would be very time-consuming and cost-intensive. A basic simulation of the point cloud with a high-fidelity sensor model, such as in [42], with a correspondingly complex scenario, is also more time-consuming and computationally intensive, as all scenes, objects, and simulation parameters have to be defined and calculated from scratch. With the augmentations, however, only a slight deviation from reality is to be expected, as the reduction in intensity in protected areas only has a small extinction coefficient and the deviation in the curvature of the scan lines only results in a slight shift in the input features of the DNNs due to the previous voxelization. However, it should be noted that synthetically generated or adjusted data may deviate to a certain extent from real data, for example, due to cross-effects between different parameters or simplifications assumed. For example, when augmenting the distance, a change in intensity is neglected, and the curvature of the scan lines is simplified. Nevertheless these limitations must be assessed depending on the application and the trade-off between accuracy and efficiency.

### 3.1. Resolution Augmentation

Two parameters in the scan pattern determine the resolution of the MEMS-based LiDAR sensor used in this work. Considering the scan pattern shown in Figure 2, the number of scan lines in the elevation direction and the number and thus the distance between two points on a scan line in the azimuth direction can be adjusted. Since the experimental setup used in this paper described in [13] does not provide any further information to increase the resolution of the point cloud by augmenting and, as shown in [13], the measurement setup has achieved a sufficient resolution of the point clouds for people detection, only the reduction in the resolution by augmentation is used. For this purpose, the azimuth and elevation resolution of the scan pattern are reduced, as described below.

#### 3.1.1. Reduction in the Azimuth Resolution

**Figure 6:**
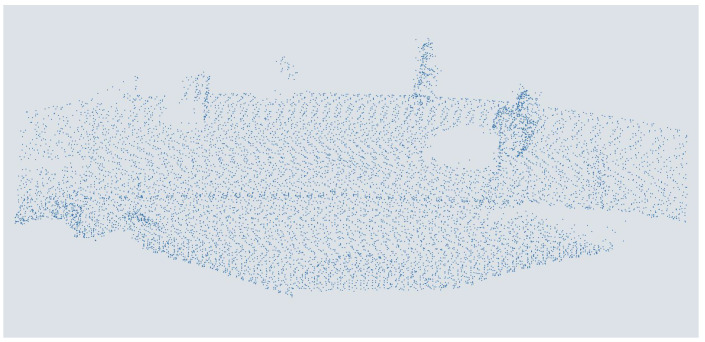
People in a point cloud augmented with one-eights azimuth resolution from a MEMS-based scanning LiDAR sensor.

For the augmentation of the data set with a lower azimuth resolution, corresponding to a sensor with a lower azimuth resolution, more and more points per scan line were deleted step by step. The points in each point cloud are assigned a point id to be identified in Python (v.3.11.6) according to their scan sequence. The number of retained points was gradually reduced for every second point scanned to achieve a continuous and efficient reduction in resolution without increasing the computational effort by using a step size that was too small. This reduced the original quantity of around 83,000 points per point cloud to as few as 20 points. This reduction in point density results in twelve new versions of the original data set, simplified in terms of point density, corresponding to an increase in the sensor distance to the object up to 26 times. The lowest resolution corresponds to the point density on an object from a sensor with a distance of about 243 m. An exemplary point cloud is shown in Figure 6.

#### 3.1.2. Reduction in the Elevation Resolution

**Figure 7:**
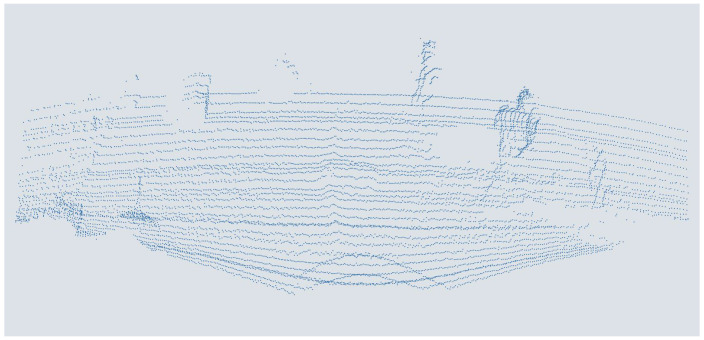
People in a point cloud augmented with one-eighth elevation resolution from a MEMS-based scanning LiDAR sensor.

For the augmentation of the data set with a low elevation resolution, corresponding to a sensor with a lower elevation resolution, an increasing number of scan lines were deleted gradually. The individual points are first assigned to the different rows to do this. This is done by determining the minima and maxima, the edge points of the scan lines, in the course of the x-values. Then, two consecutive lines are always kept except for the first one. In this way, seven augmented versions are generated from the original data sets, down to 1095 points per point cloud of the points corresponding to a point density like a 8-fold mounting height. An exemplary point cloud is shown in Figure 7.

### 3.2. Distance Augmentation

Suppose the distance between the sensor and the object is increased. In that case, the distance between the points in the point cloud and the origin of the coordinates in the sensor coordinate system increases on the one hand, and the point density on the object decreases on the other. In addition, the intensity of the measured partially reflected laser pulse decreases due to the attenuation in the atmosphere, and the scene captured by the sensor becomes larger. In order to be able to augment the influence of a greater distance between the sensor and the working area in the point cloud, the existing point cloud must be shifted from the coordinate origin in the *y*-direction, the point density on the object must be reduced, and the surrounding scene must extend to the FoV of the LiDAR sensor. The change in intensity over distance was not addressed within the scope of this work. The focus concentrated on the geometric properties, as the extinction coefficient for the weather-protected measurement setup is small, and the influence on DNNs based on point clouds, as in [43], is often negligible. This augmentation of the point cloud is discussed in more detail in the following sections.

#### 3.2.1. Reduction in the Resolution

In order to augment the distance between the sensor and object on the data set on a sensor-specific basis, the scan pattern of the Qb2 from Figure 2 must be considered. The reductions in resolution described in Section 3.1 are combined for the augmentation. As a result, the point density on the object decreases with increasing distance in both dimensions, azimuth, and elevation, corresponding to the real data. The data sets processed in this way consist of three different versions with simulated 2-fold, 4-fold, and 8-fold mounting heights or a quarter, sixteenth, and sixty-fourth of the points.

#### 3.2.2. Expanding the Measured Point Cloud

The height multiplier *H* is introduced to expand the point cloud of the surrounding scene in the sensor’s FoV. In addition to reducing the point density, the FoV, which is increased by a factor of $H^{2}$, should also be taken into account to ensure that the adjustment is as close to reality as possible, which is why new points are added around the remaining original data. The FoV is increased by a factor of $H^{2}$ to achieve a continuous and efficient increase in height without enlarging the computational effort by using a step size that was too small. A ground surface is used since no information is available for this area, which does not exist in the test area. The approximation assumes that cut-off objects and their shadows at the edge of the original FoV are not continued in the generated part. For this purpose, a reference height must be determined using an average of the existing points’ highest $y_{i}$ values. In order to extend the existing scan lines on both sides, the azimuth angles $\theta_{H,i}$ are first determined from the Cartesian $x_{i}$- and $y_{i}$-coordinates of the edge points, as described in Equation (7).

(7) $$\forall_{y}>0:\theta_{H,i}\left(x_{i},y_{i}\right)=arctan2\left(y_{i},x_{i}\right)=\left\{\begin{array}{cc} arctan(\frac{y_{i}}{x_{i}}) & \mathrm{for}\;x_{i}>0\\ arctan(\frac{y_{i}}{x_{i}})+\pi & \mathrm{for}\;x_{i}<0\\ +\frac{\pi }{2} & \mathrm{for}\;x_{i}=0 \end{array}\right.$$

Although these angles should remain maximum or minimum values, their new azimuth angle $\theta_{new}$ can be calculated by adjusting the *y*-values of the original edge points $y_{ref}$, as described in Equation (8). The two values form the limits of an interval where the azimuth angles of the points to be created are to be located. The distance should correspond to the average distance between the remaining points on the scan line.

(8) $$\theta_{new,i}=arctan2\left(y_{ref,i}\;\cdot \;tan\left(\theta_{old,i}\right),y_{ref,i}\;\cdot \;H\right)$$

In addition, complete scan lines must be added at the top and bottom, which is why the elevation angle is calculated from $z_{i}$- and $y_{i}$-values, as described in Equation (9). As with the azimuth angle, an interval can be determined in which new scan lines with the average elevation distances are inserted.

(9) $$\forall_{y}>0:\theta_{V,i}\left(y_{i},z_{i}\right)=arctan2\left(y_{i},z_{i}\right)=\left\{\begin{array}{cc} arctan(\frac{y_{i}}{z_{i}}) & \mathrm{for}\;z_{i}>0\\ arctan(\frac{y_{i}}{z_{i}})+\pi & \mathrm{for}\;z_{i}<0\\ +\frac{\pi }{2} & \mathrm{for}\;z_{i}=0 \end{array}\right.$$

With the help of $y_{ref,i}$ of the ground and *H* and the generated angles, the remaining Cartesian coordinates can be determined, as described in Equations (10) and (11).

(10) $$x_{i}=y_{ref,i}\;\cdot \;H\;\cdot \;tan\left(\theta_{H}\right)$$

(11) $$z_{i}=y_{ref,i}\;\cdot \;H\;\cdot \;tan\left(\theta_{V}\right)$$

**Figure 8:**
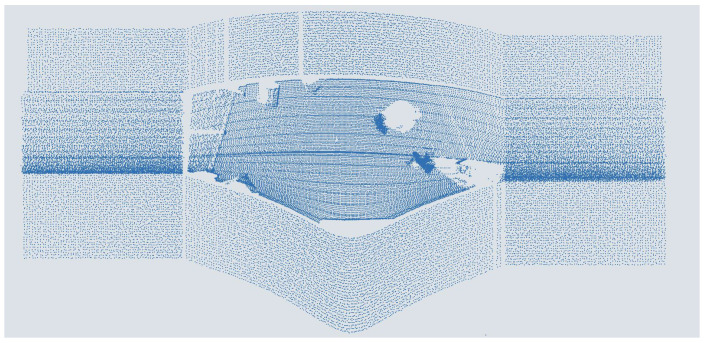
Point cloud of people augmented with double distance from a scanning ToF LiDAR sensor.

A noise function is applied to the newly generated points’ values for a more realistic arrangement. Finally, $\left(H-1\right)\;\cdot \;y_{ref,i}$ is added to the y-values of the original points and the labels to adjust the mounting height in the *y*-coordinates. The augmented point cloud thus replicates an increased mounting height of the sensor much better than a simple reduction in the point density. However, the modified data are somewhat different from real measurements. On the one hand, the scan lines are not straight but converge at the ends, as shown in Figure 2. The scan lines can be extended from the endpoints as the sensor already cuts off the intersection point. On the other hand, as shown in Figure 8, they must be continued with a constant elevation angle to avoid a too-high point density in the middle of the sides of the FoV. Also, the shadows of objects at the edge of the original area cannot be continued in the newly generated ground, and no change in intensity is assumed. These assumptions lead to deviations in the point distribution and, thus, the scan pattern in the point cloud, which can influence the prediction of the DNNs. This influence cannot be analytically precisely defined due to the black box characteristics of the DNNs. However, it can be assumed that the influence on object detection between scan patterns is transferable. This also applies to the relationship between objects at the edge of the original FoV and their shadows. As there is a consistent pattern here, there is a high probability that the DNNs learn this, which only leads to a slight deviation in the results. The deviation in the curvature of the scan lines only results in a slight shift in the input features of the DNNs due to the previous voxelization. The influence of the simplified intensity reduction can be classified as minor, as the extinction coefficient for the weather-protected measurement setup is small, and the intensity feature, for example, as in [43], has only a minor influence. However, due to the deviation, the trained models should not be transferred directly to an application; instead, they should be optimized with real data and evaluated for robustness.

### 3.3. Noise Augmentation

Figure 1 shows the working principle of a scanning TOF LiDAR sensor. To capture the 3D information in the sensor’s environment, it calculates the point of a reflection in the point cloud from the ToF, the azimuth and elevation angle of the sent laser pulse, and the intensity of the reflected laser pulse. These fundamental measurement variables can be influenced by noise. Internal sources, such as temperature variations, edge effects, and clock errors, introduce deviations in range and angular measurements. In contrast, external sources, including material properties, environmental light, and vibrations, distort signal intensity and geometry. These combined influences result in a degradation of the overall performance of the LiDAR sensor in capturing precise spatial information. Random noise probability density functions such as background radiation noise, detector noise, and circuit noise all obey Gaussian distribution, as claimed in [44]. In [45], the authors show a Gaussian noise distribution for the ToF measurement. This is why a Gaussian noise distribution is used as the basis for the augmentation of the point clouds described below. The authors do not define if this distribution has a non-zero mean, which would suggest systematic bias, or a zero mean, which would suggest entirely random noise. LiDAR noise in real-world situations should combine both, resulting from software processing, instrument imperfections, and environmental circumstances. The robustness and performance of machine learning algorithms trained may be affected if the augmentation considers only zero-mean or non-zero mean Gaussian noise, failing to capture the underlying properties of LiDAR noise. The bell-shaped curve of a Gaussian distribution, also called a normal distribution, is a continuous probability distribution [46] determined by the mean of the distribution μ and the standard deviation σ. Equation (12) gives a Gaussian distribution’s probability density function as in [46].

(12) $$f\left(x\right)=\frac{1}{\sigma \;\cdot \;\sqrt{2\;\cdot \;\pi }}\;\cdot \;e^{-\frac{{\left(x-\mu \right)}^{2}}{2\;\cdot \;\sigma^{2}}}$$

**Figure 9:**
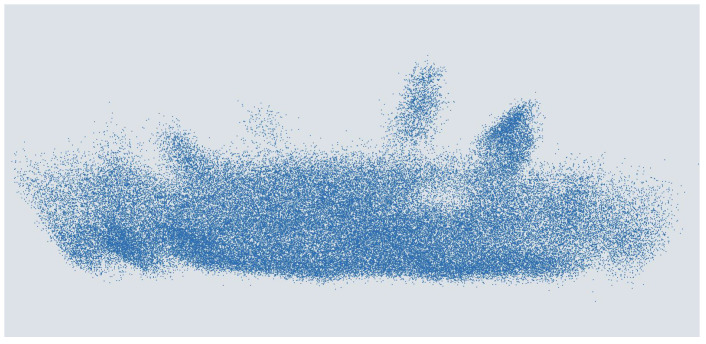
People in a point cloud augmented with Gaussian distributed noise with μ=0.3 and σ=0.04 from a MEMS-based scanning ToF LiDAR sensor.

In this work, several noise-augmented data sets are generated where different noise distributions with both μ and σ are augmented to the point clouds, as shown in Figure 9. The noise is successively increased until a test group of ten people, with different experience estimates between no experience with point clouds and the labeling of approx. 300,000 point clouds could no longer reliably distinguish people from other objects in the point clouds with the human eye. Nevertheless, all persons provide the same threshold. This threshold value is then subdivided and combined with μ to show the progression of the influence and the individual parameters. Table 1 shows the μ and σ combinations used for the augmentation of the point clouds. It should be noted that the distributions μ=0.3 and σ=0, as well as μ=0.5 and σ=0, are purely theoretical distributions that have no relevance in the technical field but are included for completeness and clarity of the evaluation.

In order to be able to assess the influence of the noise of the various measured variables of the sensor on object detection, the original data set is augmented for the parameter combinations shown in Table 1 for the geometrical values TOF, azimuth, and elevation angle of the emitted laser pulse as well as the intensity values. In addition, the combinations of noise from the angle measurement (azimuth + elevation) and the ToF and angle measurement (ToF, azimuth, and elevation) are augmented. In this way, 66 different data sets with different noise parameters are created.

### 3.4. Shading Augmentation

**Figure 10:**
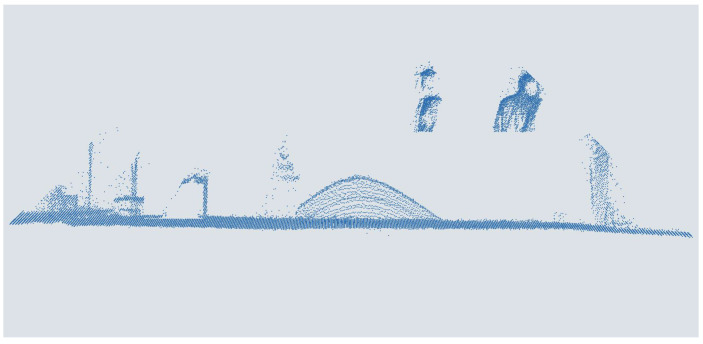
People in a point cloud augmented with 1 m shading from a MEMS-based scanning ToF LiDAR sensor.

A small distance between several people or a non-central sensor position can cause shadowing. This means that, as described in [13], other obstacles in the direction of the laser beam partially obscure people or objects. For example, very small people can disappear completely in the point cloud, while at least the head of a full-grown person is usually captured. To simulate occlusions of various sizes, points are removed directly above the ground in strips with heights ranging from 0.1 m to 1.8 m in 0.1 m steps. This achieves shading from no shading to complete occlusion of an adult of average height [47] with steadily increasing augmented shading without excessively increasing the computational effort due to a too small step size. This is shown as an example in Figure 10. The aim is to determine how much of a body must be visible to reliably detect people. The points to be removed with the corresponding *y*-values are found and deleted by sorting the data points by height.

## 4. Dataset for the Influence Analysis

Based on the measurement setup presented in [13], a data set is recorded with the Qb2 (SN: ZXRPMBIAC, Firmware: CHIARA v1.12.7) to train the DNNs. Based on this data set, different data sets with various resolutions, distances to the scene, noise, and shading of people in the point cloud of the LiDAR sensor are augmented to investigate the individual influence of the parameters on the object detection performance of DNNs. To train DNNs for people detection, the data sets are converted into the custom data set format of the OpenPCDet Toolbox described in [40]. To convert the data to the new file format, the point clouds are converted from .pcd format to .npy files with the *x*, *y*, *z*, intensity format, and the labels from JSON format to the unified 3D box definition label format for each object *i* and the maximum number of detectable classes *n* as described in [13] with ${\hat{x}}_{i}\in R,{\hat{y}}_{i}\in R,{\hat{z}}_{i}\in R,{\hat{d}}_{x_{i}}\in R,{\hat{d}}_{y_{i}}\in R,{\hat{d}}_{z_{i}}\in R,\hat{\alpha_{i}}\in \left[-\pi ,\pi \right],{\hat{c}}_{i}\in N|0\leq {\hat{c}}_{i}<n$: $\left[{\hat{x}}_{i},{\hat{y}}_{i},{\hat{z}}_{i},{\hat{d}}_{x_{i}},{\hat{d}}_{y_{i}},{\hat{d}}_{z_{i}},\hat{\alpha_{i}},{\hat{c}}_{i}\right]$.

In this way, 95 data sets with 14,819 different labeled point clouds are generated for the training and testing of the DNNs. Each data set is divided into train, test, and validation data, as shown in Table 2. A sample of each data set is publicly available at https://huggingface.co/datasets/LukasPro/PointCloudPeople (accessed on 11 March 2025) and licensed under the CC-BY-4.0.

## 5. Artificial Neural Network Training

The OpenPCDet Toolbox (v0.5.2) is used to train DNNs. The data sets are loaded as custom data sets in the configuration file, and the SECOND and PV-RCNN networks are selected for training. As it is only a matter of people detection, the configurations of both network architectures were adapted to detect people as covered in the data sets. Two different scenarios are examined during training and testing. On the one hand, the case where a network is trained with optimal data, i.e., the original data, and then tested with the augmented data, corresponds to cost-efficient sensor hardware and, on the other hand, the training and testing of the networks with the augmented data. The hyperparameters learning rate and batch size are then optimized, and both network architectures are trained for a maximum of 100 epochs each.

## 6. Results

This chapter presents the results of the DNNs SECOND and PV-RCNN training and testing with the different augmented data sets. These are presented in the following chapters depending on the augmented parameter. For each augmented data set, the DNNs are trained with the original data set, tested with the corresponding augmented data set (E), and trained and tested with the augmented data set (TE). For calculating the AP and MIoU, as described in Section 2.4, a threshold value of IoU=0.5 is used to calculate the confusion metrics, as in [39]. Both DNNs show promising results for people detection for the original non-augmented data set. The SECOND network achieves an AP=90.58% and a MIoU=0.5976 for the best epoch. The PV-RCNN achieves an AP=90.51% and a MIoU of 0.6331 for the best epoch. Thus, both networks achieve very good results for people detection for both metrics compared to the existing literature as for SECOND AP=83.13%,AP=83.34%,AP=90.97% in [36,37,48] and for PV-RCNN AP=90.25%,AP=90.25% in [37,48]. Considering the influence of the augmented effects on the prediction of the DNNs, the MIoU metric is primarily used in the further course of this work for the best possible comparability since the AP presented in [39] is only partially applicable.

### 6.1. Results for Azimuth Resolution Augmentation

**Figure 11:**
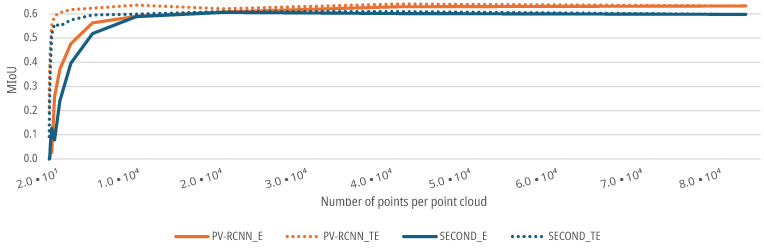
Impact of augmented azimuth resolution on the MIoU of DNNs for people detection.

For the data sets with an augmented azimuth resolution, as described in Section 3.1.1, both the SECOND and the PV-RCNN show a lower MIoU for a low azimuth resolution of the corresponding scenes of the point clouds. The initial MIoU of both DNNs of 0.5976 for the SECOND and 0.6331 for the PV-RCNN remains at a stable level for both the E- and TE-cases up to a resolution of 10,402 points in the point cloud with a degradation <5% (see Figure 11). Afterward, the MIoU of both DNNs for the E-case decreases further with decreasing azimuth resolution. For the TE-case, the MIoU of both DNNs only drops to a MIoU of 0.574 for the SECOND and 0.6177 for the PV-RCNN up to a resolution of 2601 points before it then also decreases significantly for both networks with decreasing resolution, see Figure 11. Thus, both DNNs show that the prediction accuracy decreases with decreasing azimuth resolution. For the present measurement setup or scenario, the azimuth resolution of the sensor could be reduced by a factor of 8 without significant degradation in the prediction accuracy of the DNNs. In addition, the results show that even networks trained with high resolutions can deliver good results up to a reduction in the azimuth resolution by a factor of 8, with a degradation of the MIoU of <5% with 0.5976 for the SECOND and 0.6062 for the PV-RCNN; by training the DNNs with a lower resolution, the performance can even be maintained up to a resolution reduced by a factor of 32, with a degradation of the MIoU of <5% with 0.6177 for the SECOND and 0.5745 for the PV-RCNN.

### 6.2. Results for Elevation Resolution Augmentation

**Figure 12:**
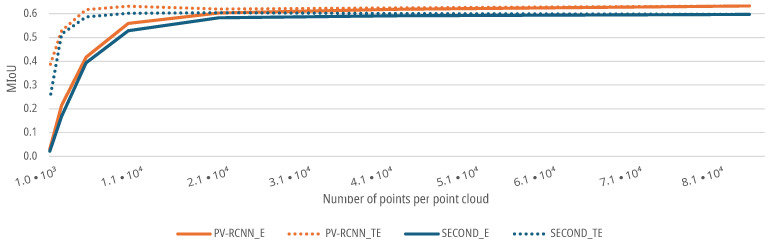
Impact of augmented elevation resolution on the MIoU of DNNs for people detection.

For the data sets with an augmented elevation resolution, as described in Section 3.1.2, both DNNs show similar behavior as the reduced azimuth resolution. The SECOND and the PV-RCNN have a lower MIoU for a low elevation resolution of the respective scenes of the point clouds. The initial MIoU of both DNNs of 0.5976 for the SECOND and 0.6331 for the PV-RCNN remains stable for both the E and TE-cases up to a resolution of 20,804 points of the point clouds with a degradation <5%, see Figure 12. Subsequently, the MIoU of both DNNs for the E-case decreases further with decreasing elevation resolution. For the TE-case, the MIoU of both networks only drops to a MIoU of 0.5865 for the SECOND and 0.6173 for the PV-RCNN up to a resolution of 5201 points before it then also decreases sharply with decreasing resolution, see Figure 12. Thus, both DNNs show that the prediction accuracy decreases with decreasing elevation resolution. For the present measurement setup or scenarios, the elevation resolution of the sensor could be reduced by a factor of 4 without significant degradation in the prediction accuracy of the DNNs. In addition, the results show that even DNNs trained with high resolutions, in this case, deliver good results up to a reduction in the elevation resolution by a factor of 4, with a deterioration of the MIoU of <5% with 0.5837 for the SECOND and 0.6035 for the PV-RCNN; by training the DNNs with a lower resolution, the performance can even be maintained up to a resolution reduced by a factor of 16, with a decrease in the MIoU of <5% with 0.6043 for the SECOND and 0.6195 for the PV-RCNN.

**Figure 13:**
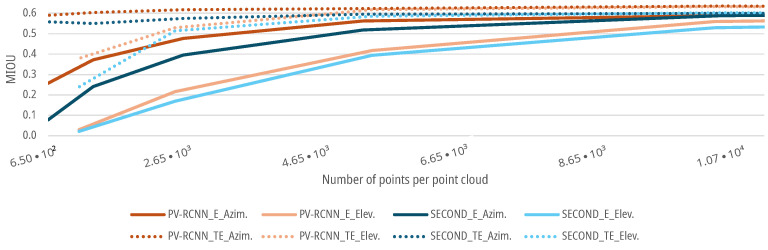
Impact comparison of augmented azimuth and elevation resolution on the MIoU of DNNs for people detection.

The following Figure 13 shows that the MIoU curves for both DNNs and training methods fall more significantly for a decrease in the elevation resolution than for a reduced azimuth resolution. This means that for the present measurement setup, the elevation resolution has a more significant influence on the people detection of the DNNs than the azimuth resolution of the sensor. The reason for this cannot be explained analytically due to the black box behavior of the DNNs. However, the deviation could be due to the data set, as groups of people who know each other tend to walk closer next to each other instead of behind each other and, therefore, need less resolution in the measurement setup in the elevation direction. Furthermore, this could also be due to the voxelization of the input features. The voxels in the implementation of the OpenPCDet Toolbox have different resolutions in the longitudinal, lateral and height directions. The voxels in the height direction, which are primarily influenced by the elevation resolution, are larger, and the resolution is therefore lower. This can lead to a cross-influence of the elevation direction augmentation on the prediction quality in the models or measurement setup used here. A further detailed analysis of this correlation should take place in the future and is discussed in Section 9.

### 6.3. Results for Distance Augmentation

**Figure 14:**
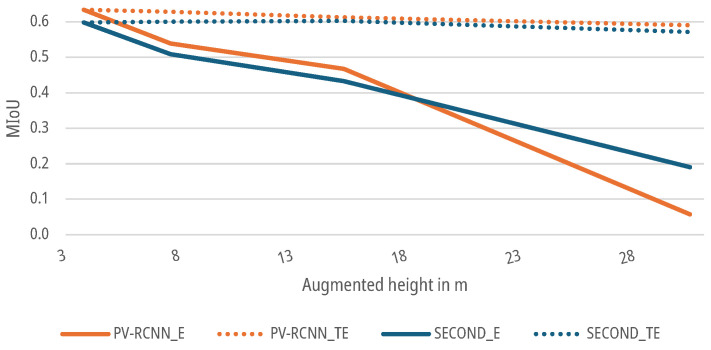
Impact of augmented height on the MIoU of DNNs for people detection.

For the data sets with an augmented higher mounting height, as described in Section 3.2, both DNNs SECOND and PV-RCNN were again trained and tested for both the E-case and TE-case. As shown in Figure 14, the MIoUs of both DNNs for the E-case drop to a MIoU of 0.0576 for the SECOND and 0.1904 for the PV-RCNN up to an augmented height of 30.4 m. The networks already fall below the 5% threshold from the first measurement point when the distance of the original measurement setup is doubled. For the TE-case, the MIoUs of both networks drop only marginally to a MIoU of 0.5712 for the SECOND and 0.5897 for the PV-RCNN at a working distance of 30.4 m. The networks do not exceed the 5% threshold up to the measurement point at a measurement height of 15.2 m with 0.6027 for the SECOND and 0.6121 for the PV-RCNN compared to the initial data sets.

### 6.4. Results for Noise Augmentation

**Figure 15:**
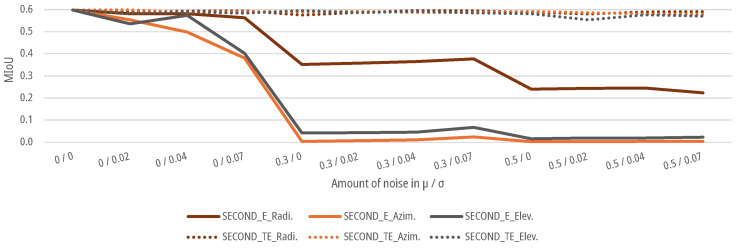
Impact of augmented noise on the MIoU of SECOND for people detection.

**Figure 16:**
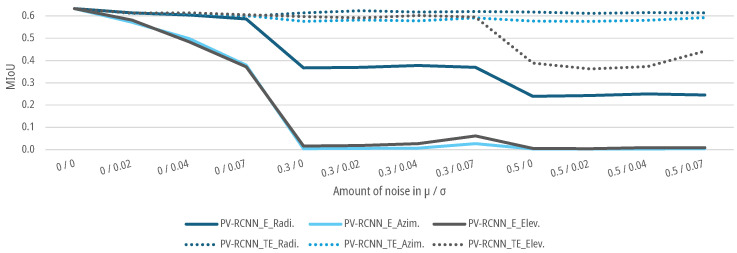
Impact of augmented noise on the MIoU of PV-RCNN for people detection.

For the data sets with an augmented noise on the geometrical parameters of the point clouds, as described in Section 3.3, DNNs SECOND and PV-RCNN are again trained and tested for the E-case and TE-case. As shown in Figure 15 and Figure 16, the MIoUs of both DNNs for the E-case drop to a MIoU of 0.0041 for azimuth noise and 0.0229 for elevation noise for the SECOND and 0.0044 for azimuth noise and 0.0087 for elevation noise for the PV-RCNN up to an augmented noise of μ=0.5 and σ=0.07. The DNNs fall below the 5% threshold for noise with a μ=0 and σ=0.04 with 0.4982 for azimuth noise and 0.574 for elevation noise for the SECOND and 0.4987 for azimuth noise and 0.4842 for elevation noise for the PV-RCNN. For the radial noise, both DNNs for the E-case drop to a MIoU of 0.2230 for the SECOND and 0.2448 for the PV-RCNN up to an augmented noise of μ=0.5 and σ=0.07. The DNNS fall below the 5% threshold for noise with a μ=0 and σ=0.07 for the SECOND with 0.5632 and for the PV-RCNN with 0.5866. In the E-case, both DNNs show significantly less sensitivity to noise in the radial measurement than in the azimuth or elevation measurement. For the TE-case, the MIoUs of both DNNs drop only marginally to a MIoU of 0.5903, 0.5792, and 0.5707 for radial, azimuth, or elevation noise for the SECOND and 0.6139, 0.5927, and 0.3619 for radial, azimuth, or elevation noise for the PV-RCNN. This means that the DNNs can learn the task of people detection on data with noise on the radial, azimuth, or elevation measurement without significant degradation. The results of both models in the E-case show that μ has a more substantial effect on the models than the σ of the noise. This could be due to the voxelization of the deep learning methods. Due to the lower effective resolution and the averaging, σ is already relativized in the data processing before the actual DNN. At the same time, a μ propagates into the network and can only be compensated there to a limited extent. This is, as shown in Figure 15 and Figure 16, critical for the E-cases, as the model cannot compensate for the bias due to the lack of training.

**Figure 17:**
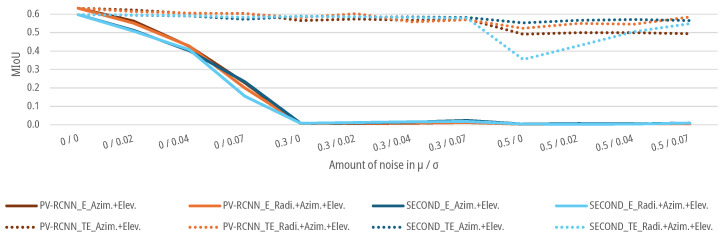
Impact of combined augmented noise on the radial, azimuth, and elevation measurement on the MIoU of DNNs for people detection.

In addition, noise is combined for the azimuth and elevation measurements as well as the radial, azimuth, and elevation measurements. In that case, the object detection performance of both models drops more sharply for both cases with slight noise of the measurement variables up to μ=0.3 and σ=0.02 than with a single noisy measurement variable, as shown in Figure 17. No significant deviations can be recognized for higher noise in the TE case. Both DNNs can learn the correlations from the data for both test cases. However, from a noise level of μ=0.5, both models show slight deterioration in learning the correlations for a combined noise level.

**Figure 18:**
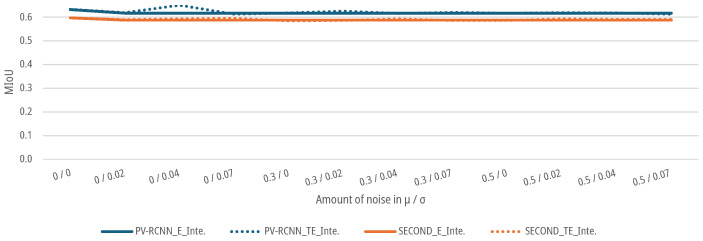
Impact of augmented noise on the intensity parameters on the MIoU of DNNs for people detection.

As shown in Figure 18, both the SECOND and the PV-RCNN show no sensitivity for the data sets augmented with noise on the intensity parameters of the point cloud. Both DNNs show for the E-case a drop to a MIoU of 0.5883 for the SECOND and 0.6173 for the PV-RCNN up to an augmented noise of μ=0.5 and σ=0.07. The prediction accuracy of the DNNs does not fall below the 5% threshold. For the TE-case, the MIoUs of both networks drop only marginally to a MIoU of 0.5909 for the SECOND and 0.6131 for the PV-RCNN at an augmented noise of μ=0.5 and σ=0.07. Again, the networks do not exceed the 5% threshold compared to the initial data sets.

### 6.5. Results for Shading Augmentation

**Figure 19:**
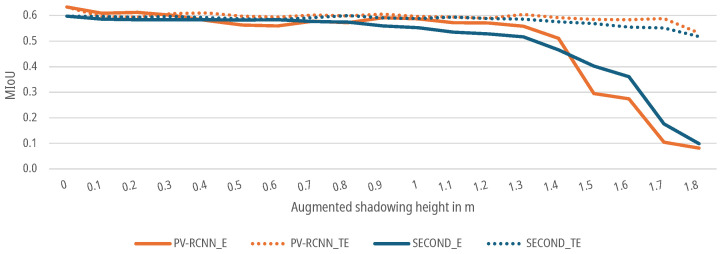
Impact of augmented shadowing on the MIoU of DNNs for people detection.

For the augmented data sets, which show the effect of shading as described in Section 3.4, both the SECOND and the PV-RCNN for the E-case as well as the TE-case, as shown in Figure 19, show a continuous decrease in the MIoU with increasing augmented shading. In the E-case, the MIoU of the predictions of both DNNs drops only marginally between 0 m and 1.3 m augmented shading to a MIoU of 0.5164 for the SECOND network and a MIoU of 0.5858 for the PV-RCNN. In the range from 1.4 m to 1.8 m, the MIoU then drops significantly to 0.0989 for the SECOND and 0.0814 for the PV-RCNN. The 5% threshold is undercut by the SECOND at 0.9 m with 0.5596 and for the PV-RCNN at 0.3 m with 0.6012. For the TE-case, the MIoU of the predictions of both DNNs drops only marginally up to a shading height of 1.7 m to a MIoU of 0.5517 for the SECOND and a MIoU of 0.5884 for the PV-RCNN. The 5% threshold is achieved at an augmented shading height of 1.6 m for the SECOND with 0.5549 and at 0.5 m for the first time with 0.5977 and 1.4 m with 0.5913 for the PV-RCNN. In the range from 1.7 m to 1.8 m, the MIoU of both DNNs begins to drop significantly. For the test subjects with heights in this range, the retrained networks show that they can detect people only with very few points and their shadows on the ground plane.

## 7. Optimum Number of Sensors and Sensor Position

**Figure 20:**
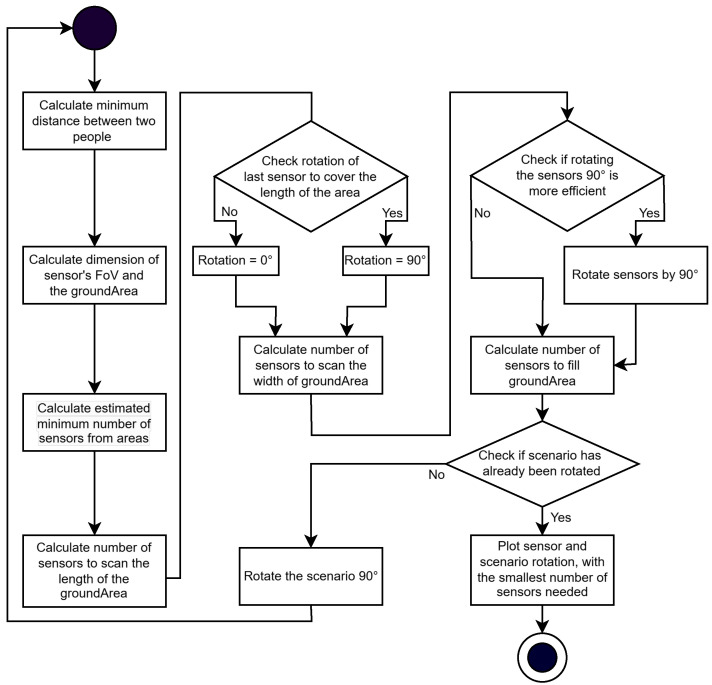
Flowchart of the program calculating the minimum number of sensors for a defined working area.

In order to cover a working area as cost-effectively and efficiently as possible, a minimum number of sensors is required, as the sensors account for a significant proportion of the costs of the entire measurement setup. In [13], the calculation of a sufficient ceiling height is shown to ensure that a sensor detects at least one point of a person detection between the 5% and 95% percentile of people’s heights in Germany at typical distances between people. The overlap of the FoVs of different sensors is not considered since according to [13], this reduces the efficiency of the measurement setup without guaranteeing reliability. This approach is combined with the results from the previous Section 6.5, where a maximum shadowing on 1.3 m can be accepted with a degradation of less than 5% MIoU in the TE case. The TE case was selected because it is based on implementing a real measurement setup, and the models are presumably trained based on their existing data. Looking at a test group with a mean height of 1.73 m [47], combined with the results of the maximum shadowing of 1.3 m, a minimum height of 0.5 m from the body of the person detected in the FoV of the sensors for the given people height distribution can be defined, which the used DNNs needs, in order to detect people reliably without being explicitly trained on shadowing. Thus, 0.5 m is defined as the minimum height from which a person can be detected for the program to optimize the number of sensors and, compared to [13], to calculate the optimized number of sensors for detection at object detection level and not at point level. This method is used to develop a program that allows the optimal arrangement of LiDAR sensors to be found for work areas of different sizes. This allows the minimum number of sensors to be determined to save on costs and resources. For this purpose, the sensor parameters horizontal angle FoV $\left(FoV_{h}\right)$, vertical angle FoV $\left(FoV_{v}\right)$, the mounting height *h*, and the length *l* and width *b* of the working area are program inputs. Afterward, a plot with the optimized positioning of the sensors above the working area for detection at the point cloud level and object detection level is generated. The program calculates the optimum sensor alignment, as shown in Figure 20.

**Figure 21:**
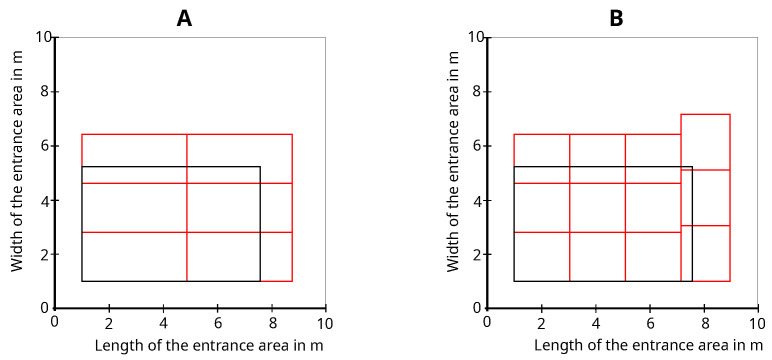
Optimized sensor number and orientation for detection at point cloud level (**A**) and detection on object detection level (**B**) based on the measurement setup.

The measurement setup-dependent maximum permissible FoV is calculated from the minimum scan height of 0.5 m for shadowed people detection and the minimum distance between two people $d_{min}=0.45\;m$ and height difference $d_{g}$ described in [49], as shown in Equation (13), as the basis for optimizing the number of sensors and orientation. Based on the example of the measurement setup used to generate the data set, this optimization process results in the number of sensors and sensor orientation shown in Figure 21A for detection at the point cloud level and Figure 21B for detection at the object list level. The black rectangle shows the given working area, and the red rectangles show the detection areas for each sensor. The smaller detection area in Figure 21B for detection at the object list level resulting from the minimum height without shadowing a person for an object detection compared to detection at point cloud level in Figure 21A is recognizable.

(13) $$FoV=2\;\cdot \;\arctan\left(\frac{d_{min}}{d_{g}}\right)$$

## 8. Conclusions

This article investigates the influence of different LiDAR sensors and mounting parameters on the people detection performance of DNNs by augmenting a real data set. For this purpose, 95 different data sets are generated with the different sensor and mounting parameters resolution, noise, scene distance, and shadowing of people detections. Their influence on the people detection performance of different state-of-the-art DNNs is compared. Real scenarios are processed and evaluated through the hardware-oriented augmentation of the original data set for people detection performance. The results show that the resolution of the sensors can be reduced and the working distance increased within the measurement setup used in this project with only minor degradation on object list level of less than 5% with a reduced resolution of up to 32 factors or a distance of 4 factors. In addition, a higher noise level can be tolerated in the geometric and intensity measurements with a degradation on object list level of less than 5% with a Gaussian noise up to μ=0 and σ=0.07. The results show that the tested DNNs are more sensitive to reduced elevation resolution than the azimuth resolution. This could be due to the information extraction in the DNNs, which partially compensates for a widespread distribution, whereas the shift in the entire distribution also leads to a shift in the information. Due to the black box characteristics of the DNNs, a fully analytical approach is not feasible here either. In addition, the networks show a higher sensitivity for noise in the azimuth and elevation measurement than for the ToF measurement. For the shading of people, it is shown that 0.5 m should be mapped in the point cloud from one point downwards to guarantee reliable people detection. Based on this information, a software program is presented that determines the minimum number of sensors required and their position based on the parameters of the measurement setup for detections at both point cloud and object detection levels. The results enable optimizing the sensor systems and the entire measurement setup to make LiDAR people detection systems more efficient, cost-effective, and reliable.

## 9. Outlook

Based on the results presented in this paper, LiDAR sensors can be developed more specifically for people counting and security applications in the future. Using the results obtained, sensor manufacturers can reduce costs and increase the sensors’ energy efficiency through adapted sensor technology. The augmentation methods presented can also enrich data sets and thus contribute to developing and training more robust DNNs. In addition, the insights gained into shadowing and the software for optimizing the number of sensors in an overall measurement setup can further contribute to the further spread of LiDAR-based people counting and security applications on the market and thus further improve reliability and data protection in the future. In future work, the relationship between the combinations of the parameters considered separately here, as well as the combination of different model configurations, such as the resolution of the voxelization and the object detection should be investigated, as this will certainly lead to crossover effects. This is possible, for example, when examining the combination of an increased distance and a lower resolution. The increased distance already reduces the point density on a measurement object, which influences object detection quality. An even lower resolution of the sensor would reduce the point density on the object even further and further impair object detection quality. The same effects could also occur with noise and a lower point density on the measurement object, for example, due to a lower resolution or a greater distance.

## Figures and Tables

**Table 1:** Augmented parameters of Gaussian distributions.

μ	0	0	0	0.3	0.3	0.3	0.3	0.5	0.5	0.5	0.5
** σ **	0.02	0.04	0.07	0	0.02	0.04	0.07	0	0.02	0.04	0.07

**Table 2:** Train, test, and validation datasplit.

Dataset	Size
Train	10,373
Test	3111
Validation	1334

## Data Availability

A section of the original data presented in the study are openly available at https://huggingface.co/datasets/LukasPro/PointCloudPeople (accessed on 11 March 2025) and licensed under the CC-BY-4.0.
